# FIT, a regulatory hub for iron deficiency and stress signaling in roots, and FIT-dependent and -independent gene signatures

**DOI:** 10.1093/jxb/eraa012

**Published:** 2020-01-10

**Authors:** Birte Schwarz, Petra Bauer

**Affiliations:** 1 Institute of Botany, Heinrich Heine University, Universitätsstr. 1, Düsseldorf, Germany; 2 Cluster of Excellence on Plant Science (CEPLAS), Heinrich Heine University, Düsseldorf, Germany; 3 University of Missouri, USA

**Keywords:** ABA, bHLH, cell elongation, co-expression, FIT, iron, protein interaction, subgroup Ib, subgroup IVb, subgroup IVc

## Abstract

Iron (Fe) is vital for plant growth. Plants balance the beneficial and toxic effects of this micronutrient, and tightly control Fe uptake and allocation. Here, we review the role of the basic helix–loop–helix (bHLH) transcription factor FIT (FER-LIKE FE DEFICIENCY-INDUCED TRANSCRIPTION FACTOR) in Fe acquisition. FIT is not only essential, it is also a central regulatory hub in root cells to steer and adjust the rate of Fe uptake by the root in a changing environment. FIT regulates a subset of root Fe deficiency (–Fe) response genes. Based on a combination of co-expression network and FIT-dependent transcriptome analyses, we defined a set of FIT-dependent and FIT-independent gene expression signatures and co-expression clusters that encode specific functions in Fe regulation and Fe homeostasis. These gene signatures serve as markers to integrate novel regulatory factors and signals into the –Fe response cascade. FIT forms a complex with bHLH subgroup Ib transcription factors. Furthermore, it interacts with key regulators from different signaling pathways that either activate or inhibit FIT function to adjust Fe acquisition to growth and environmental constraints. Co-expression clusters and FIT protein interactions suggest a connection of –Fe with ABA responses and root cell elongation processes that can be explored in future studies.

## Teaser

FIT-dependent gene signatures and FIT protein interactions highlight links to developmental and stress signaling, and suggest a connection with abscisic acid responses and root cell elongation.

## Introduction

The micronutrient iron (Fe) is a main cofactor for electron transfer reactions. Fe shuttles between the ferric (Fe^3+^) and ferrous (Fe^2+^) states during oxidation and reduction. Fe-dependent redox reactions occur in many different biochemical processes in plants, ranging from energy generation and photosynthesis to various primary, secondary, and hormone biosynthetic pathways (Connorton *et al.*, 2017). Although Fe is among the most prevalent elements on Earth, the amount of bioavailable Fe is limited due to its complexation in insoluble Fe hydroxides. Calcareous and alkaline conditions further decrease bioavailable Fe concentrations by several magnitudes (Colombo *et al.*, 2014). Therefore, plants actively mobilize Fe in the soil, and different strategies evolved for that. Most plant species secrete small molecules into the rhizosphere to chelate soil Fe^3+^, namely carboxylic acids, coumarins, and riboflavin derivatives; they acidify the rhizosphere and reduce Fe^3+^ to Fe^2+^ (Strategy I). Poaceae chelate Fe^3+^ by phytosiderophores of the mugineic acid family (Strategy II), whereby lowland rice in submerged soils also uses Strategy I (Marschner and Römheld, 1994; Kobayashi *et al.*, 2014; Connorton *et al.*, 2017). In this way, Strategy I plants take up Fe^2+^, while Strategy II plants import mainly Fe^3+^–phytosiderophore chelates.

Fe malnutrition is spotted in plants because of resulting leaf coloration phenotypes. Leaf chlorosis is a typical Fe deficiency (–Fe) symptom, with pale to yellow leaf color in intercostal leaf areas particularly pronounced in young small remaining leaves. Chloroplast differentiation and chlorophyll biosynthesis, Fe cofactor-dependent processes, are compromised in the chlorotic leaves. Ultimately, plants grow and reproduce poorly. Autophagy may take place in older leaves to remobilize Fe (Avila-Ospina *et al.*, 2014). On the other hand, leaf bronzing is a sign of Fe toxicity. Excessive Fe is deleterious, and free Fe reacts with reactive oxygen species (ROS), that are often produced in response to stress factors. Fe^3+^/Fe^2+^ react in the chemical Haber–Weiss and Fenton reactions with superoxide anions, hydrogen, and lipid peroxides, generating hydroxyl and lipid alkoxyl radicals. Cells have mechanisms based on antioxidant compounds and enzymes to protect from different ROS species. However, the very toxic hydroxyl and lipid alkoxyl radicals are not usually eliminated by cellular functions. These radicals damage biomolecules non-specifically, and cause DNA mutations and defective proteins (Le *et al.*, 2019).

The demand for Fe changes during development from vegetative to seed filling and senescence (Pottier *et al.*, 2019). Environmental challenges such as high light, drought periods, or flooding drastically disturb growth rates and growth physiology, and alter requirements for Fe. Restricting the uptake of reactive metal ions (Dubeaux *et al.*, 2018) alleviates toxic effects of radical species, which occur under such stresses.

Root cells perceive and interpret developmental and environmental signals to adjust Fe uptake and Fe homeostasis. The transcription factor FIT (FER-LIKE FE DEFICIENCY-INDUCED TRANSCRIPTION FACTOR) plays a predominant role in sustaining and restricting the amount of Fe in plant roots of eudicots (Colangelo and Guerinot, 2004; Jakoby *et al.*, 2004; Yuan *et al.*, 2005). Arabidopsis FIT, like characterized orthologs, tomato FER and rice OsbHLH156 (Ling *et al.*, 2002; Wang *et al*., 2020), are basic helix–loop–helix (bHLH) family proteins and members of the bHLH subgroup IIIa+c (Pires and Dolan, 2010). FIT is activated upon –Fe by a bHLH transcription factor cascade in roots (Gao *et al.*, 2019*b*). bHLH121/URI of the bHLH subgroup IVb (Gao *et al.*, 2019*a*; Kim *et al.*, 2019), together with bHLH subgroup IVc transcription factors, bHLH034, bHLH104, bHLH105/ILR3, and bHLH115, stimulate directly, by promoter binding, the transcription of several –Fe response genes including subgroup Ib *BHLH* genes in roots and in shoots (Zhang *et al.*, 2015; Li *et al.*, 2016; Liang *et al.*, 2017; Wang *et al.*, 2017; Tissot *et al.*, 2019). In rice, subgroup IVb protein OsbHLH064 is as yet uncharacterized, and bHLH IVc factors OsbHLH057/PRI4, OsbHLH058/PRI2, OsbHLH059/PRI3, and OsbHLH060/PRI1 are regulators of Fe uptake (Zhang *et al.*, 2017; Kobayashi *et al.*, 2019; Zhang *et al*., 2020). bHLH subgroup Ib transcription factors, bHLH038, bHLH039, bHLH100, and bHLH101, then interact with FIT to activate FIT (Yuan *et al.*, 2008; Wang *et al.*, 2013). In tomato, this regulatory module is represented by subgroup Ib SlbHLH068 and FER (Du *et al.*, 2015), whereas in rice it is represented by IRO2 and OsbHLH156 (Wang *et al*., 2020). Activated FIT, tomato FER, and rice OsbHLH156 promote expression of a subset of root-specific –Fe response genes, acting in root Fe acquisition. Grafting studies with the tomato *fer* mutant showed that this gene function is essential only in roots, where these genes are predominantly expressed (Brown *et al.*, 1971).

An informative co-expression network of FIT-dependent and FIT-independent processes has been useful in Arabidopsis (Ivanov *et al.*, 2012). The ATTED tool that served to generate this network has in the meantime been updated to include RNA sequencing (RNA-seq) transcriptome data (Obayashi *et al.*, 2018). *fit* mutant transcriptome data also became available (Mai *et al.*, 2015, 2016).

Root Fe uptake is sensitive to environmental and developmental influences, and FIT plays a major role in this regulation. FIT activity is fine-tuned through protein–protein interactions involving several key regulators of hormone and stress response pathways.

Here, we provide in the first part of this review a comprehensive updated framework for the co-expression network including *FIT*, highlighting FIT-dependent and FIT-independent gene signatures and gene functions. In the second part, we discuss transcriptional and post-translational control mechanisms that steer FIT activity and integrate signals from multiple signaling pathways. FIT is thus part of a central root cell regulatory hub, that receives developmental and environmental information to adjust Fe uptake and homeostasis. ABA signaling and root cell elongation may also control FIT action.

## Update and applications of FIT-dependent and FIT-independent co-expression networks

**Fig. 1.**
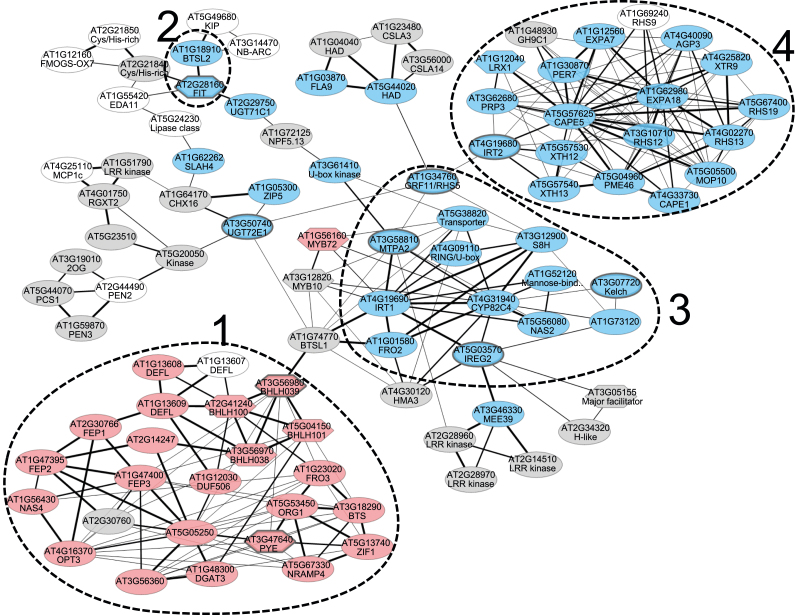
FIT-dependent and FIT-independent co-expression network generated with a subset of root-expressed Fe deficiency (–Fe)-induced genes in *Arabidopsis thaliana.* The network combines co-expression analysis of selected –Fe-regulated genes with expression profiles using a *fit* mutant transcriptomic study. The network was generated using the ATTED-II tool, version 9.2 (Obayashi *et al.*, 2018) that integrates RNA-seq data from the DNA Data Bank of Japan (DDBJ), and processed with Cytoscape. Thickness of edges corresponds to expression correlation based on the ATTED-II Mutual Rank scoring system. Robust –Fe root marker genes At3g07720, *UGT72E1*, *BHLH039*, *IRT2*, and *MTPA2* (Ivanov *et al.*, 2012), and *IREG2*, *GRF11*, *PYE*, and *FIT* were used as query genes (marked by thick lines). The chosen query genes allow generation of a comprehensive framework with cluster 1–4 genes, including *FIT* in addition to the majority of previously represented genes in the network (compare with Ivanov *et al.*, 2012). The color code indicates regulation behavior according to comparative transcriptomic data sets of *fit* mutant plants (*fit-3* T-DNA insertion loss-of-function allele; Jakoby *et al.* 2004; Bauer *et al.* 2007; Mai *et al.*, 2015, 2016) versus the wild type. Transcriptomic data had been produced with 6-day-old seedlings, grown on vertical half-strength Hoagland agar plates with 50 µM (+) Fe or without (–) Fe, and 6-week-old roots of plants, raised in hydroponic culture with quarter-strength Hoagland medium with 10 µM Fe, that had been transferred for 1 week to medium with 10 µM (+) Fe or without (–) Fe [Mai *et al.* (2016) additional file 3, Dataset 1, 6-week-old roots, and Dataset 2, 6-day-old seedlings, in both column D, *fit* –Fe versus WT –Fe, and column G, WT –Fe versus WT +Fe]. Red, up-regulated in (WT –Fe/WT +Fe) and up-regulated in (*fit* –Fe/WT –Fe) in at least one of the data sets; blue, up-regulated in (WT –Fe/WT +Fe) and down-regulated in (*fit* –Fe/WT –Fe) in at least one of the data sets; gray, other/no regulation (e.g. expression <1.5-fold ratio threshold); white, not included in the transcriptomic data set. Dashed lines encircle four clusters. Cluster 1 genes are induced by –Fe in roots and shoots and are FIT-independent. Cluster 2–4 genes are induced by –Fe in roots and are FIT-dependent, whereby cluster 2 and 3 genes are mostly FIT-dependent in 6-day-old seedlings and in 6-week-old roots, and cluster 4 genes mostly in 6-week-old roots. The list of co-expressed genes and their regulation patterns are available in Supplementary Table S1.

Many physiological responses to –Fe are the result of altered gene expression regulation. In Arabidopsis wild-type plants, ~1300 genes are up-regulated and 1250 genes down-regulated in different spatio-temporal zones of the root response during exposure to –Fe, representative of different functional categories, such as hormonal and developmental, physiological, and stress pathways (Dinneny *et al.*, 2008; Schwarz *et al*., 2020). Loss-of-function mutants of the transcription factor FIT [*fit-3*, exon T-DNA insertion allele, (Bauer *et al.*, 2007)] express Fe uptake genes at a low level in roots, and mobilize and take up insufficient amounts of Fe; for example, they have low expression of *IRT1* and *FRO2*, low root Fe reductase activity, and low Fe contents in leaves and seeds (Jakoby *et al.*, 2004; Gratz *et al.*, 2019). Consequently, these *fit* mutant plants grow poorly and display severe leaf chlorosis. Robust marker genes are defined as consistently regulated by –Fe across different growth conditions applied in different laboratories (Ivanov *et al.*, 2012). Robust marker genes At3g07720, *UGT72E1*, *IRT2*, and *MTPA2* are expressed at a low level in *fit* mutant roots, while the robust marker gene *BHLH039* is highly induced (Ivanov *et al.*, 2012; Mai *et al.*, 2016). *fit* mutant global transcriptome data (*fit-3* allele) have been obtained for 6-day-old seedlings exposed to + and –Fe and for roots dissected from 6-week-old plants exposed for a week to + and –Fe, using modified Hoagland’s medium (Mai *et al.*, 2015, 2016). Gene co-expression information using ATTED-II (Obayashi *et al.*, 2018) can be integrated in combination with the aforementioned transcriptomic data of the regulatory *fit* mutant (Mai *et al.*, 2016) to illustrate co-expression links and nodes of regulated genes in a comprehensive manner. In this way, distinct subsets of –Fe-regulated genes, highlighting FIT-dependent and FIT-independent –Fe response clusters, become apparent (Fig. 1; Supplementary Table S1 at *JXB* online). Four co-expression clusters represent meaningful –Fe response signatures that are applied to place new genetic components into hierarchical order in order to reconstruct regulatory pathways and circuits. In the following, we focus on genes in these four clusters.

**Fig. 2.**
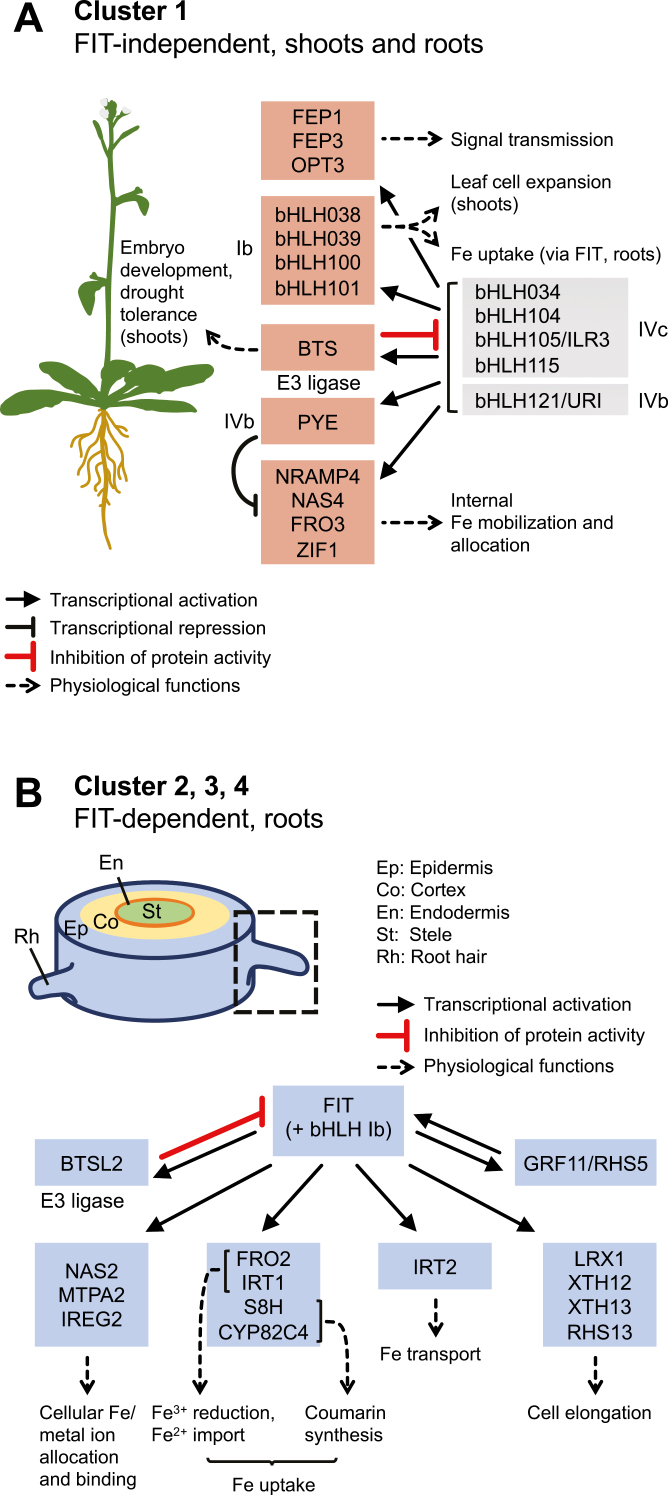
Cellular functions encoded in the co-expression clusters 1–4. (A) Cluster 1 genes code for proteins that activate the FIT pathway, regulate Fe homeostasis, allocate Fe and other metal ions to shoots and roots, or play other roles in plant shoots and roots. Cluster 1-encoded proteins are bHLH038, bHLH039, bHLH100, bHLH101 of bHLH subgroup Ib transcription factors; NRAMP4, Fe transporter; NAS4, nicotianamine synthase; FRO3, ferric chelate reductase; ZIF1, zinc-nicotianamine transporter; OPT3, Fe (signal) transporter; FEP1, FEP3, small proteins; PYE, bHLH subgroup IVb transcription factor; and BTS, E3 ubiquitin ligase. Other proteins depicted are transcription factors bHLH034, bHLH104, bHLH105/ILR3, and bHLH115 of bHLH subgroup IVc, bHLH121/URI of subgroup IVb, and FIT of bHLH subgroup IIIa+c. (B) Cluster 2, 3, and 4 genes code for components of root Fe uptake, cellular metal homeostasis, and root cell elongation in the root epidermis (epidermis indicated by a rectangle). Cluster 2-encoded proteins are FIT, bHLH transcription factor, and BTSL2, E3 ubiquitin ligase. Cluster 3-encoded proteins are S8H, scopoletin hydroxylase; CYP82C4, fraxetin hydroxylase; FRO2, ferric reductase; IRT1, Fe^2+^ transporter; GRF11/RHS5, 14-3-3 protein; MTPA2, zinc transporter; IREG2, Fe/metal transporter; and NAS2, nicotianamine synthase. Cluster 4-encoded proteins are IRT2, Fe^2+^ transporter; LRX1, leucine-rich repeat and extensin protein; XTH12, XTH13, xyloglucan endotransglucosylases; amd RSH13, root hair-specific. Other proteins depicted are bHLH subgroup Ib transcription factors. Further information on cluster 1–4 genes is provided in the text, Fig. 1, and Supplementary Table S1.

Cluster 1 is a FIT-independent cluster, induced by –Fe in 6-day-old seedlings and in 6-week-old plant roots and shoots also in the absence of FIT (24 genes, up-regulated at –Fe versus +Fe in the wild type and up-regulated in *fit-3* versus the wild type at –Fe, in at least one of the data sets, seedlings and/or roots, according to Mai *et al.* (2016); marked in red in Fig. 1; summary of their protein functions in Fig. 2A; see also the list in Supplementary Table S1). These genes are responsive to internal –Fe signals from shoots and roots. Cluster 1 genes are mostly targets of bHLH proteins of the subgroups IVb and IVc (Zhang *et al.*, 2015; Li *et al.*, 2016; Liang *et al.*, 2017; Wang *et al.*, 2017; Gao *et al.*, 2019*a*; Kim *et al.*, 2019; Tissot *et al.*, 2019) or down-regulated by bHLH011 and PYE (Long *et al.*, 2010; Tanabe *et al.*, 2019). At least one cluster 1 gene, *NAS4*, is up-regulated by MYB10 and MYB72 (Palmer *et al.*, 2013) (MYB10 and MYB72 in gray and red color, Fig. 1). Cluster 1-encoded proteins regulate the allocation of Fe and other metals, activate FIT and Fe uptake, and play roles in embryo and leaf development or in drought responses (see references below). Subgroup Ib proteins bHLH038, bHLH039, bHLH100, or bHLH101 bind and activate FIT protein, as mentioned in the Introduction (Wang *et al.*, 2007; Yuan *et al.*, 2008; Wang *et al.*, 2013). They also regulate leaf cell expansion and the transition of photosynthesis in leaves (Andriankaja *et al.*, 2014). The metal chelator nicotianamine, generated by nicotianamine synthases, such as NAS4, enables Fe allocation to young sink tissues (Schuler *et al.*, 2012). Mitochondrial ferric chelate reductase FRO3 (Jain *et al.*, 2014), vacuolar Fe exporter NRAMP4 (Lanquar *et al.*, 2005; Bastow *et al.*, 2018), and vacuolar metal–nicotianamine transporter ZIF1 (Haydon *et al.*, 2012) allocate Fe and metal ions in cells and in the plant. Phloem transporter OPT3 transmits Fe or a shoot-derived Fe signal long distances to roots (Zhai *et al.*, 2014). FEP1 and FEP3 are small proteins (<5 kDa, also called peptides), possibly mobile in the phloem, that activate Fe uptake (Grillet *et al.*, 2018; Hirayama *et al.*, 2018). A bHLH transcription factor of the subgroup IVb, PYE, negatively regulates some of the cluster 1 functions, as mentioned above (Long *et al.*, 2010). BTS is an E3 ligase with a hemerythrin-like domain and di-Fe-oxygen-binding sites. BTS binds and affects protein abundance of bHLHIVc proteins (Long *et al.*, 2010; Selote *et al.*, 2015; Liang *et al.*, 2017), similar to rice HRZ1 (Kobayashi *et al.*, 2013). It was proposed that –Fe is sensed through BTS E3 ligase stability, affecting subgroup IVc bHLH transcription factor levels (Kobayashi *et al.*, 2013; Selote *et al.*, 2015; Rodriguez-Celma *et al*., 2019*a*).

Two closely related BTS-like proteins, BTSL1 and BTSL2, are also likely candidates as negative regulators (Hindt *et al.*, 2017; Rodriguez-Celma *et al*., 2019*b*). *BTSL1* is placed just in between clusters 1 and 3, as it is not FIT dependent (not differentially regulated in *fit-3* versus the wild type, at + or –Fe; Mai *et al.*, 2016; Fig. 1; Supplementary Table S1) but induced by –Fe versus +Fe in roots.

A characteristic of cluster 2, 3, and 4 genes is FIT dependency together with root-specific expression [up-regulated at –Fe versus +Fe in the wild type, down-regulated in *fit-3* versus the wild type at –Fe, according to Mai *et al.* (2016); marked in blue in Fig. 1; summary of their protein functions in Fig. 2B; see also the list in Supplementary Table S1]. Induction of cluster 2–4 genes in roots is also under the control of shoot-derived signals. Shoot to root communication is, however, not the topic of this review (Mendoza-Cozatl *et al*., 2014; Zhai *et al.*, 2014). *BTSL2* is found together with *FIT* in a designated cluster 2, which forms a regulatory circuit. BTSL2 enhances FIT protein degradation in plant cells (Rodriguez-Celma *et al*., 2019*b*). Cluster 3 is the FIT-dependent ‘root Fe uptake’ cluster, and most of its genes are induced by FIT in both 6-day-old seedlings and roots of 6-week-old plants (13 genes; Figs 1 and 2B; Supplementary Table S1). Fe is acquired via the action of coumarins, synthesized among others by the enzymes S8H and CYP82C4, via Fe reductase FRO2 and the Fe^2+^ importer IRT1 (Eide *et al.*, 1996; Robinson *et al.*, 1999; Vert *et al.*, 2002; Rajniak *et al.*, 2018; Siwinska *et al.*, 2018; Tsai *et al.*, 2018). The 14-3-3 protein GRF11 stimulates Fe acquisition responses and *FIT* induction, possibly via nitric oxide signals (Yang *et al.*, 2013). Vacuolar metal transporters, MTPA2 and IREG2/FPN2, and nicotianamine synthase NAS2 mediate metal homeostasis of root epidermis cells (Arrivault *et al.*, 2006; Schaaf *et al.*, 2006; Morrissey *et al.*, 2009; Schuler *et al.*, 2012). Cluster 4 is a FIT-dependent cluster (19 genes; Figs 1, 2B), whereby most of its genes are dependent on FIT in the roots of 6-week-old plants, while they are not induced under –Fe in seedlings (Supplementary Table S1). Nutrition affects the development of roots (Gruber *et al.*, 2013). Root morphological changes in response to –Fe include typically the elongation and branching of root hairs, differentiation of transfer cells, and root architectural changes with an elongation of the main root, as documented in many plant species (Ling *et al.*, 2002; Schikora and Schmidt, 2002*a*, *b*; Gratz *et al.*, 2019). Root morphological phenotypes in response to –Fe can vary with respective growth conditions, particularly in Arabidopsis (Gruber *et al.*, 2013; Wild *et al.*, 2016). Cluster 4 shows that FIT is required for inducing root cell elongation genes. Root elongation is a developmental process that occurs in response to –Fe in a FIT-dependent manner in Hoagland’s medium, a condition which was used for the transcriptomic study (Ling *et al.*, 2002; Mai *et al.*, 2015, 2016; Gratz *et al.*, 2019). The Fe transporter gene *IRT2* is a tandem duplicate of *IRT1*, but cannot complement loss of *IRT1*, perhaps due to predominant localization of IRT2 in endosomes instead of the plasma membrane (Vert *et al.*, 2009). LRX1 is an extracellular leucine-rich repeat and extensin protein that functions in root hair morphology (Baumberger *et al.*, 2001). XTH12 and XTH13 are members of a family of cell wall-modifying xyloglucan endotransglucosylases presumably functioning in cell elongation (Maris *et al.*, 2011). Likewise, RSH13 is an extracellular protein for cell wall integrity and root hair cell elongation under phosphate deficiency and ethylene treatment (Tanaka *et al.*, 2017).

**Fig. 3.**
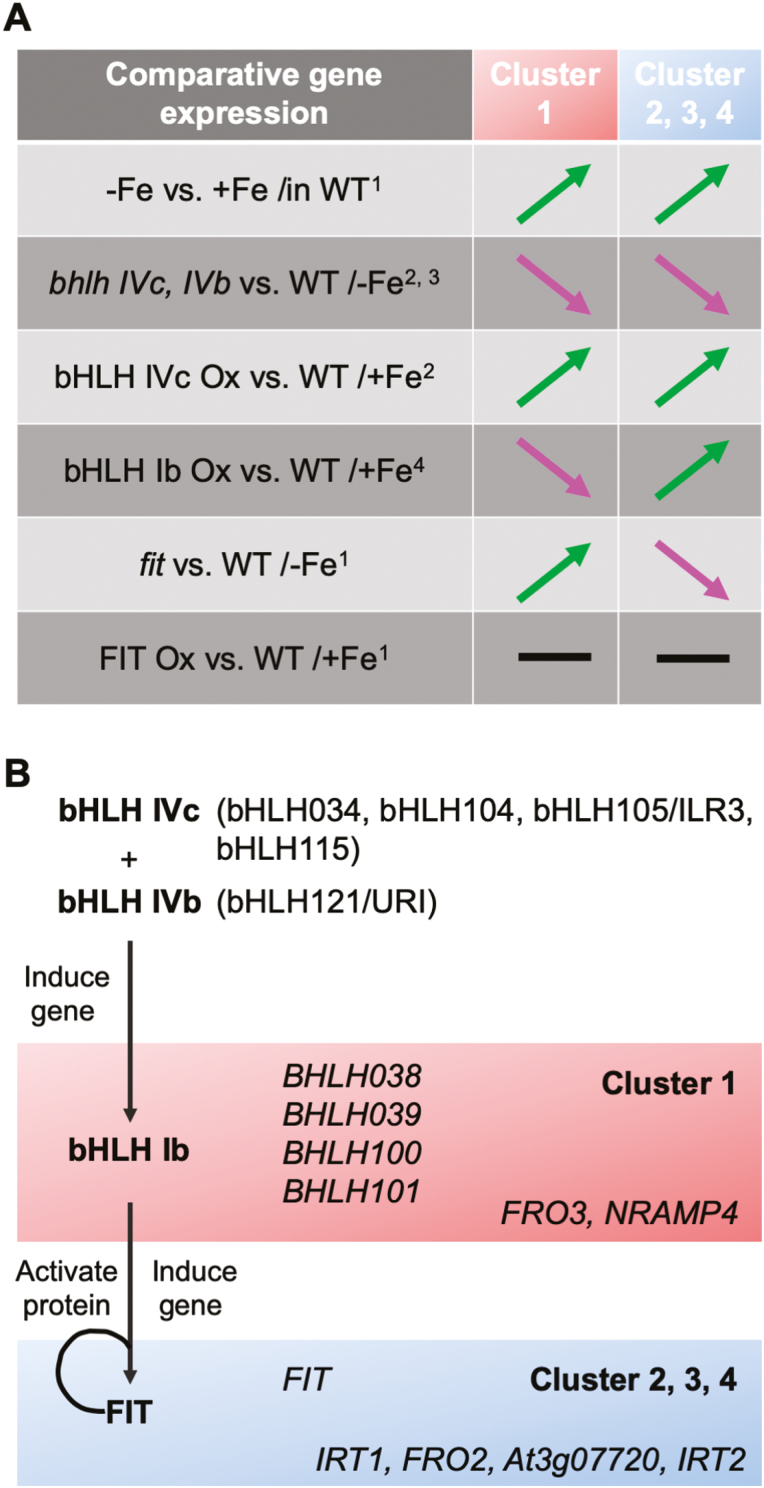
Use of co-expression clusters to integrate genetic components in a hierarchical model of Fe deficiency (–Fe) response regulation. (A) Summary of FIT-independent cluster 1 and FIT-dependent cluster 2, 3, and 4 gene regulation in the respective comparative analyses: ^1^(Mai *et al.*, 2016), ^2^(Zhang *et al.*, 2015; Li *et al.*, 2016; Tissot *et al.*, 2019), ^3^(Gao *et al*., 2019*a*; Kim *et al.*, 2019), and ^4^(Naranjo-Arcos *et al*., 2017); green and pink arrows, up- and down-regulated in the comparison; black trait, not regulated. (B) bHLH transcription factor cascade regulating cluster 1–4 genes in response to –Fe. bHLH subgroup IVc proteins and bHLH121/URI bHLH IVb protein bind to the promoters of *BHLH* subgroup Ib genes and other cluster 1 genes. bHLH subgroup Ib proteins interact with and activate FIT to steer induction of cluster 2, 3, and 4 genes. A feedback from Fe signals to bHLH factors is not indicated in the scheme. Characteristic example marker genes are noted. Further information on cluster 1–4 genes is provided in the text, Fig. 1, and Supplementary Table S1.

Information on the co-expression clusters 1–4 has served to integrate genetic components for –Fe regulation in a conclusive manner into a hierarchical model of –Fe response regulation (Fig. 3). Reduced activity of bHLH subgroup IVc proteins and knockout of bHLH121/URI eliminates induction of cluster 1, 2, 3, and 4 genes at –Fe, while their overactivation can stimulate genes of all four clusters at sufficient Fe (+Fe) despite Fe overaccumulation (Zhang *et al.*, 2015; Li *et al.*, 2016; Gao *et al.*, 2019*a*; Kim *et al.*, 2019; Tissot *et al.*, 2019). A stimulation of genes from all four clusters together with Fe overload in the shoot is also a phenotype of mutants lacking a suppressive long-distance +Fe signal from the shoot, such as *opt3* and nicotianamine-deficient mutants (Schuler *et al.*, 2012; Khan *et al.*, 2018). On the other hand, overexpression of the bHLH Ib protein bHLH039 leads to Fe accumulation in shoots and decreased expression of cluster 1 genes. Cluster 2, 3, and 4 genes are induced in bHLH039 overexpression plants, in line with enhanced Fe acquisition (Naranjo-Arcos *et al*., 2017). Obviously, high-level expression of cluster 1 genes is not a prerequisite for shoot Fe overaccumulation, rather cluster 1 genes are responsive to –Fe signals. This is confirmed in Fe-deficient *fit* mutants that fail to activate cluster 2, 3, and 4 genes at –Fe, while cluster 1 genes are highly up-regulated. On the other hand, FIT overexpression does not lead to a major cluster 1–4 gene expression change, and, along with the absence of an Fe accumulation phenotype at +Fe, this indicates that FIT protein is not functional at +Fe (Mai *et al.*, 2016). This supports that FIT is activated by bHLH subgroup Ib proteins. These subgroup Ib proteins respond to the –Fe signal cascade, while *BHLH* subgroup Ib genes are not targets of FIT.

Taken together, the concept of deriving gene expression signatures from the co-expression network with emphasis on clusters 1–4 has proven to be reliable for determining the hierarchy of regulatory factors in a transcriptional regulation cascade. The co-expressed genes of Supplementary Table S1 comprise an ‘–Fe response’ term for gene functional enrichment studies. The gene signatures explain regulated physiological and cellular pathways leading to Fe acquisition and metal homeostasis.

## Transcriptional and post-translational control mechanisms of FIT to integrate hormone and stress signals

**Fig. 4.**
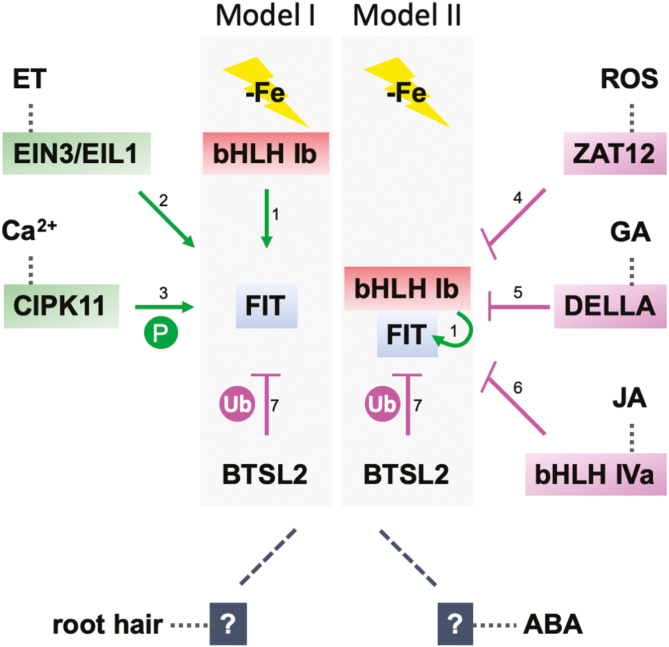
FIT as a regulatory hub for integrating signals from multi-parallel signaling pathways. The proteins, indicated in the model, interact directly via protein–protein interactions with FIT in plant cells (indicated by solid lines) to adjust Fe acquisition in response to the respective signals. Root hair development and ABA are prospective signals for FIT regulation, as highlighted in this review; however, interacting proteins remain to be identified (indicated by dashed lines and question marks). The light gray boxes indicate two models for interaction, either protein interactions only with FIT (Model I, left side) or protein interactions with the FIT–bHLH subgroup Ib protein complex (Model II, right side). Abbreviations used are –Fe, Fe deficiency; ET, ethylene; ROS, reactive oxygen species; GA, gibberellic acid; JA, jasmonic acid; P, phosphorylation; Ub, ubiquitination; ABA, abscisic acid. Green color, positive effect; pink color, negative effect; dark gray color, positive or negative effect not yet determined; red color, cluster 1-encoded; blue color, cluster 2-encoded. References are ^1^(Yuan *et al.*, 2008; Wang *et al.*, 2013), ^2^(Lingam *et al.*, 2011), ^3^(Gratz *et al*., 2019), ^4^(Le *et al.*, 2016), ^5^(Wild *et al.*, 2016), ^6^(Cui *et al.*, 2018), and ^7^(Rodriguez-Celma *et al*., 2019).

The ability to adjust Fe acquisition to internal and external stimuli is crucial for plants during developmental phase transitions and fluctuations of environmental stress conditions. Protein complexes of key regulators cross-connect signaling pathways. Such integration hubs for multi-parallel signaling pathways are, for example, DELLA, PIF4, and EIN3, that together steer cell elongation (Choi and Oh, 2016). FIT is controlled at the post-translational level through protein interactions (Wu and Ling, 2019). Besides its bHLH domain, FIT possesses N- and C-terminal subdomains that mediate interactions with other proteins and are partly even sufficient for the protein–protein interactions. Most known protein interactions of FIT involve its C-terminal subdomain. Because of its self-activating activity, full-length FIT protein fused with a DNA-binding domain could not be used to screen for novel protein interactors in the yeast two-hybrid assay. Instead, the C-terminal subdomain of FIT fused with a DNA-binding domain was fully sufficient to successfully retrieve novel interactors (Lingam *et al.*, 2011;  Gratz *et al*., 2019, 2020). Subsequently, full-length FIT protein–protein interactions are validated through targeted yeast two-hybrid interaction assays with swapped DNA-binding and activation domains, plant bimolecular fluorescence complementation (BiFC), plant Förster resonance energy transfer (FRET) acceptor photobleaching, plant FRET-fluorescence lifetime imaging (FLIM), and *in vitro* and/or plant cell co-immunoprecipitation (co-IP) (Yuan *et al.*, 2008; Lingam *et al.*, 2011; Wang *et al.*, 2013; Le *et al.*, 2016; Wild *et al.*, 2016; Cui *et al.*, 2018; Gratz *et al*., 2019, 2020; Rodriguez-Celma *et al*., 2019*b*). In the following, we discuss that FIT activity is responsive to signals from multi-parallel signaling pathways. FIT is thus part of a regulatory hub, which steers Fe acquisition in a positive and negative manner (Fig. 4).

*FIT* is expressed in roots where it is induced 2- to 3-fold at –Fe versus +Fe (Colangelo and Guerinot, 2004; Jakoby *et al.*, 2004). FIT itself promotes its own gene induction in a feed-forward loop together with a bHLH subgroup Ib protein (cluster 2 gene), and this is a major explanation for the –Fe transcriptional induction of *FIT* (Wang *et al.*, 2007; Naranjo-Arcos *et al*., 2017). Interestingly, *FIT* gene induction levels are higher in the absence of polycomb repressive complex PRC2-mediated histone 3 lysine 27 trimethylation, and this methylation pathway acts predominantly on cluster 2 and 3 genes as well as root growth at –Fe (Park *et al.*, 2019). The PRC2 methylation complex also mediates methylation in environment- and stress-responsive genes, including abscisic acid (ABA)-responsive genes (Kleinmanns *et al.*, 2017). The promoter of one component of the complex is targeted by bHLH subgroup IVc protein bHLH105/ILR3 in yeast (de Lucas *et al.*, 2016). However, this interaction was not validated *in planta*, and therefore it remains unknown whether ILR3 can activate or repress the PRC2 complex. The subgroup IVb protein bHLH011 negatively regulates *FIT* and cluster 3 gene expression, but positively affects cluster 1 genes including *BHLH* subgroup Ib genes (Tanabe *et al.*, 2019). Presently, it cannot be explained why *FIT* expression is down-regulated although subgroup Ib genes are up-regulated. On the other hand, *BHLH011* is down-regulated in response to –Fe and in bHLH039 overexpression plants, suggesting that bHLH011 could be an –Fe repressor (Naranjo-Arcos *et al*., 2017; Tanabe *et al.*, 2019). Future studies will address how –Fe signals are perceived to down-regulate *BHLH011* and to regulate the PRC2 methylation complex. The connection between bHLH105/ILR3 and expression of PRC2 components will be interesting to explore.

FIT protein is most drastically controlled at the post-translational level, which includes proteasomal degradation, phosphorylation, cellular localization, mobility, and the interactions with activating and inhibiting proteins (see references below). In six reported overexpression lines, FIT protein is detected at +Fe and –Fe, and physiological studies clearly demonstrate that overexpressed FIT or tomato FER are not active at +Fe (Jakoby *et al.*, 2004; Brumbarova and Bauer, 2005; Meiser *et al*., 2011; Sivitz *et al.*, 2011; Gratz *et al.*, 2019). Higher FIT protein abundance at +Fe compared with –Fe was also partly noted, although not quantified (Meiser *et al*., 2011; Sivitz *et al.*, 2011).

As described above, FIT is activated by interaction with bHLH subgroup Ib transcription factors, bHLH038, bHLH039, bHLH100, and bHLH101, at –Fe (Yuan *et al.*, 2008; Wang *et al.*, 2013). bHLH039 is reported to exert the strongest effect among them on –Fe response regulation, and is often selected as representative of the subgroup Ib (Sivitz *et al.*, 2012; Wang *et al.*, 2013; Maurer *et al.*, 2014). bHLH subgroup Ib proteins are not induced at +Fe. This explains why FIT remains inactive when it is overexpressed (Jakoby *et al.*, 2004; Meiser *et al*., 2011; Gratz *et al.*, 2019). bHLH039 cannot trigger cluster 2, 3, and 4 activation on its own (Naranjo-Arcos *et al*., 2017). bHLH039 together with FIT, but neither one alone, induce the target *IRT1* promoter in plant, yeast, and mammalian cells, and in transgenic plants, showing that bHLH039 and FIT are a functional module in cells (Yuan *et al.*, 2008; Wild *et al.*, 2016; Gratz *et al.*, 2020). bHLH039 fused with a fluorescent protein is rather immobile and localizes close to the cell periphery and to only a small extent in the cell nucleus (Trofimov *et al*., 2019). Its localization is drastically shifted to the nucleus when bHLH039 is co-expressed together with FIT (Trofimov *et al*., 2019). Similarly, IRO2 localization is shifted from the cytoplasm to the nucleus in the presence of bHLH156 (Wang *et al*., 2020). The localization of FIT itself does not change in the absence or presence of bHLH039 (Trofimov *et al*., 2019). Hence, not only is FIT activated by bHLH subgroup Ib proteins, but also vice versa. Generally, bHLH proteins dimerize via their HLH domains. Formation of the FIT–bHLH039 heterocomplex also involves the C-terminal domain of FIT, and the interaction preferentially takes place when FIT is mutated at Ser272 to provide a phospho-mimicking site, but not when it is mutated in this way at Tyr237 and Tyr278 (Gratz *et al*., 2019, 2020). The mutant FIT form mimicking Ser272 phosphorylation shows a higher nucleus to cytoplasm ratio of localization than wild-type FIT (Gratz *et al*., 2019, 2020). It is therefore most likely that FIT protein is present in an inactive state, unless bHLH subgroup Ib protein is expressed. Upon serine phosphorylation, FIT interacts better with bHLH subgroup Ib protein, and the complex is partitioned more towards the nucleus, where *trans*-activation of cluster 2–4 genes takes place.

A small pool of FIT protein is present in phosphorylated form in plant roots, which coincides with a small pool of active FIT protein, while a large pool of non-phosphorylated FIT protein represents inactive FIT (Gratz *et al.*, 2019). The serine protein kinase CIPK11 phosphorylates FIT at Ser 272 and activates FIT (Gratz *et al.*, 2019). *CIPK11* is induced by –Fe, but it is not a FIT target. CIPK11 is also activated under –Fe by calcium signaling and interacts with calcium sensors CBL1 and CBL9, needed for full-level –Fe response regulation via FIT (Gratz *et al.*, 2019). CBL–CIPK complexes decode calcium signaling in response to abiotic stress. Drought, ABA, and alkaline soil conditions affect the ability of plants to take up Fe, and CIPK11 acts as a regulator in these responses with dual roles. CIPK11 phosphorylates and activates ABI5, but on the other hand it phosphorylates Cys2/His-type zinc finger transcription factor Di19-3, resulting in down-regulation of some drought responses (Zhou *et al.*, 2015; Ma *et al.*, 2019). CIPK11 also phosphorylates and inactivates the H^+^-ATPase AHA2, involved in rhizosphere acidification, thus negatively affecting proton extrusion (Fuglsang *et al.*, 2007; Yang *et al.*, 2019). AHA2 activity is important for Fe acquisition in alkaline and calcareous conditions. More studies are needed to resolve the apparently contrasting roles of CIPK11 in these different stress situations. Similarly, another calcium-regulated CIPK, CIPK23, has promoting and inhibitory roles in Fe uptake regulation by targeting FRO2 and IRT1 proteins (Tian *et al.*, 2016; Dubeaux *et al.*, 2018). In these cases, a combination of –Fe with metal excess but not –Fe alone could trigger CIPK23 activation. More than one serine is targeted by CIPK11 in FIT, and FIT is probably the target of more than one serine protein kinase (Gratz *et al.*, 2020). Other potential candidate protein kinases are mitogen-activated protein kinase 3 (MAPK3) and MAPK6, that are induced by –Fe and stimulate cluster 2 and 3 genes (Ye *et al.*, 2015). Multiple protein kinases may thus transmit multiple phosphorylation marks, and the sum of protein phosphorylation may represent the signal to fine-tune the activity of FIT. In nature, complex regulation is needed for optimal acclimation. Open questions relate to the mechanism and importance of transcriptional *CIPK11* and CIPK11 protein activation at –Fe as well as the role of specific CBL–CIPK decoders. Spatial information is lacking about the CIPK11 activation by CBL1/9 under –Fe and about the phosphorylation of FIT by CIPK11. Importantly, the possible role of CIPK11 in connecting abiotic stress and –Fe signaling needs to be further elucidated.

Fe uptake and FIT activity are promoted by ethylene and nitric oxide, and both signaling molecules, often associated with stress responses in plants, stabilize FIT protein (Lingam *et al.*, 2011; Meiser *et al*., 2011). The ethylene-induced transcription factors EIN3/EIL1 increase FIT protein abundance through interaction with FIT (Lingam *et al.*, 2011). EIN3/EIL1 also prevent photo-oxidative stress (Zhong *et al.*, 2009). Several downstream targets of EIN3/EIL1 upon –Fe are photo-oxidative stress-induced genes (Lingam *et al.*, 2011). Such genes are also misregulated in triple *bhlh* subgroup Ib mutant seedlings (Maurer *et al.*, 2014). Even though FIT function and root Fe acquisition are not perturbed, leaf developmental phenotypes are found in the triple mutants (Andriankaja *et al.*, 2014; Maurer *et al.*, 2014). Besides increasing Fe acquisition in the context of photo-oxidative stress, EIN3/EIL1 may also regulate root hair growth in conjunction with FIT under –Fe (Feng *et al.*, 2017).

Target gene induction is preceded by the assembly of the transcription factor complex at an enhancer region and bending of the DNA towards RNA polymerase II, bridged by the large multiprotein complex Mediator. FIT interacts with Mediator subunit MED16, while EIN3/EIL1 interact with MED25, both being subunits of the Mediator tail that attaches to the transcription factor complex (Yang *et al.*, 2014; Zhang *et al.*, 2014). *med16* and *med25* mutants have lower expression of cluster 2, 3, and 4 genes. Consequently, they have lower Fe contents and their leaves are more chlorotic than wild-type leaves when plants are exposed to –Fe. Thus, the effect of MED16 and MED25 can be explained through an activation of –Fe responses via EIN3/EIL1 and FIT (Yang *et al.*, 2014; Zhang *et al.*, 2014). Interestingly, the composition of the Mediator complex varies in response to developmental and environmental cues. MED25 also suppresses ABA responses (Chen *et al.*, 2012) and acts on hydrogen peroxide-mediated root hair cell elongation (Sundaravelpandian *et al.*, 2013), amongst other responses (Buendia-Monreal and Gillmor, 2016). An interesting question to answer in the future is whether and how the Mediator tail is informed about –Fe signals.

FIT activity is suppressed by protein interactions with ZAT12, DELLA, jasmonate-induced bHLH proteins, and BTSL2. In these cases, the inhibition of FIT may be an advantage to avoid toxic Fe effects or to deprive cells of Fe for optimal growth. ZAT12 is a C_2_H_2_-type zinc finger nuclear protein induced by ROS in response to multiple abiotic stresses (Davletova *et al.*, 2005). *ZAT12* expression is under control of bZIP29 (Ben Daniel *et al.*, 2016). bZIP29 plays a role in cell proliferation and cell wall organization in the root meristem. A dominant-negative form of bZIP29 caused an increased expression of several cluster 1, 2, and 3 –Fe genes, which could be linked to the increased root meristem and wavy root phenotypes (Van Leene *et al.*, 2016). *ZAT12* is induced by a prolonged exposure to –Fe. In this condition, hydrogen peroxide is produced (Le *et al.*, 2016). Interestingly, hydrogen peroxide generation in roots is dependent on FIT, and FIT protein is more stable when treated with hydrogen peroxide, dependent on the presence of ZAT12 (Le *et al.*, 2016). Since EIN3/EIL1 and ZAT12 are both EAR-motif-containing transcription factors, they may compete for FIT binding. Under high Fe conditions, high levels of hydrogen peroxide are generated, and *ZAT12* is highly induced (Le *et al.*, 2016, 2019). *zat12* mutants do not show an apparent high Fe phenotype, which could be due to the redundancy of ZAT proteins (Xie *et al.*, 2019). Likewise, we are lacking more information on the relevance of ZAT12 in coordinating Fe acquisition in response to abiotic stress.

The DELLA repressor protein of gibberellic acid signaling accumulates in the root meristem to inhibit root growth, and it is able to interact directly with FIT (Wild *et al.*, 2016). Using transgenic reporter lines with native promoter and transgenic promoter swapping experiments, the authors were able to develop a spatially resolved model for the action of DELLA protein on FIT and the regulation of Fe uptake. According to this model, DELLA protein is degraded under –Fe in the root epidermis of the root differentiation zone, so that FIT remains active. On the other hand, DELLA that accumulates in the root epidermis interacts with the FIT bHLH subgroup Ib complex and prevents its target DNA promoter binding (Wild *et al.*, 2016).

Jasmonic acid (JA) causes proteasomal degradation of FIT and possibly of bHLH subgroup Ib proteins. This explains the negative effect of JA supply on Fe acquisition and Fe contents (Maurer *et al.*, 2011; Kobayashi *et al.*, 2016; Cui *et al.*, 2018). The four redundant JA-induced bHLH subgroup IVa transcription factors, bHLH018, bHLH019, bHLH020, and bHLH025, interact with FIT. FIT is inactive in this complex and is degraded more quickly (Cui *et al.*, 2018). JA also increases the biosynthesis of secondary compounds. In some plant species, bHLH subgroup Ib proteins are induced by JA, resulting in secondary compound biosynthesis (Goossens *et al.*, 2017). bHLH020 is required for methyl-JA-induced formation of endoplasmic reticulum (ER) bodies, associated with defense compound release upon herbivory in Brassicaceae (Matsushima *et al.*, 2004; Nakazaki *et al.*, 2019). Overexpression of bHLH025 results in enlarged roots and cotyledons, increased lateral root number, and higher susceptibility to cyst nematode infection (Jin *et al.*, 2011). In other plant species, bHLH IVa proteins regulate secondary compound biosynthesis, relevant in the context of plant defense (Goossens *et al.*, 2017). The connection between JA and Fe may highlight a mechanism of controlling Fe acquisition and Fe homeostasis upon an infection with pathogens and herbivores (Romera *et al.*, 2019). Recent studies showed that bHLH subgroup IVb and IVc proteins regulate glucosinolates under –Fe and that this affects herbivore susceptibility (Samira *et al.*, 2018).

FIT is degraded via the proteasome (Sivitz *et al.*, 2011; Meiser *et al*., 2011; Gratz *et al.*, 2020), possibly mediated by the E3 ubiquitin ligase BTSL2 (Rodriguez-Celma *et al*., 2019*b*). BTSL2 promotes FIT degradation under –Fe in plants (Rodriguez-Celma *et al*., 2019*b*), but it is unclear why BTSL2 does not enhance degradation of FIT upon shoot Fe overload although cluster 2–4 genes including *BTSL2* are induced (Zhang *et al.*, 2015; Naranjo-Arcos *et al*., 2017). Perhaps a different E3 ligase is even more important to directly ubiquitinate FIT protein in plants, and this as yet unknown E3 ligase may be ineffective upon overexpression of bHLH regulators. The role of BTSL1 remains elusive, although from its similarity and partial redundancy with BTSL2 it was concluded that it may also target FIT (Rodriguez-Celma *et al*., 2019*b*). Generally, BTS/BTSL1/BTSL2 data need to be treated with caution. BTS/BTSL proteins are very unstable, and difficult to study at the biochemical level in plant cells, so that a majority of conclusions about their cellular protein interactions and biochemical roles remain to be proven in statistically validated and controlled protein interaction plant cell experiments (Selote *et al.*, 2015; Rodriguez-Celma *et al*., 2019*b*).

Lastly, tyrosine phospho-mimicking within the C-terminal domain of FIT negatively affects FIT function in cells and in Arabidopsis. Phospho-mimicking of FIT at Tyr238 and Tyr278 resulted in less partitioning towards the nucleus, less interaction with bHLH039, less *trans*-activation of target genes, and partly enhanced degradation of FIT in plant protein extracts (Gratz *et al.*, 2020). Hence, a presently unknown regulatory pathway involving a tyrosine kinase might steer FIT activity. Such a pathway could involve a MAPK kinase kinase, such as Raf10, which targets ABI5, the same transcription factor target as CIPK11 (Nguyen *et al.*, 2019). Brassinosteroid signaling also involves tyrosine kinase activities (Belkhadir and Jaillais, 2015). In future studies, it will be interesting to identify tyrosine kinase candidates regulating Fe uptake.

## Conclusions and outlook

The here-presented co-expression network is a comprehensive framework for Fe regulation studies, highlighting selected but meaningful co-expressed gene functions that are representative for responses of physiological and cellular pathways. The co-expression clusters reflect FIT-dependent and FIT-independent regulation mechanisms downstream of the bHLH transcription factor cascade. The combined information about co-expression and FIT-dependent regulation in one network helps researchers to select marker genes and understand their expression patterns. The network eases the characterization of novel regulatory mechanisms and mutants with Fe acquisition and Fe homeostasis phenotypes by combining mutant analysis or stress acclimation studies with comparative transcriptome analysis to understand the hierarchies of regulatory events. This similar type of co-expression network with FIT-dependent and FIT-independent co-expression clusters has also been applied to uncover putative *cis*-regulatory promoter elements and transcription factor-binding sites, allowing prediction of transcriptional regulators of FIT-dependent and FIT-independent genes in addition to bHLH transcription factors (Schwarz *et al*., 2020). More research is required to further characterize transcriptional regulation pathways and to pay more attention to cell and tissue specificity. *BHLH* subgroup IVc promoters are predominantly active in the root stele (de Lucas *et al.*, 2016; Samira *et al.*, 2018), similar to several cluster 1 genes (Wang *et al.*, 2007; Long *et al.*, 2010). However, the regulation of FIT and cluster 2–4 genes takes place mainly in the root epidermis (Jakoby *et al.*, 2004). Transcription factors and regulatory components may be mobile from the inner root stele to the root epidermis. More research is needed to investigate this mobility and the mechanisms behind it, and investigate the dependency on structural requirements, post-translational modifications, and protein interaction complexes, also at the level of transcription factor partitioning within the cell.

FIT may be the sole target in regulatory hubs in the root, but it may also be the complex of FIT with a bHLH subgroup Ib protein (model I versus model II in Fig. 4), as shown for the FIT–DELLA interaction (Wild *et al.*, 2016). Competitive effects between different types of interactors may also be conceivable. Several FIT protein interactors are activated by –Fe in roots (e.g. CIPK11 and Mediator), but the mechanisms that perceive and transduce –Fe signals to control them (*CIPK11* gene induction and Mediator protein assembly) remain unknown. Signaling molecules such as calcium, ROS, ethylene, and nitric oxide may be very important.

Several FIT-interacting proteins act in ABA responses and root cell elongation, suggesting close molecular connections of these signaling pathways with FIT (Fig. 4, indicated by dashed lines). ABA is generated in response to –Fe in the apoplast, and exogenously applied ABA enhances depletion of apoplastic Fe. In this way, ABA alleviates –Fe leaf chlorosis (Lei *et al.*, 2014). Like FIT, ABI5 is a target of CIPK11 (Zhou *et al.*, 2015; Gratz *et al.*, 2019). MED25 and the polycomb complex for transcriptional activation also have connections with ABA signaling (Kazan, 2017; Kleinmanns *et al.*, 2017). Besides the cluster 4 target genes of FIT, multiple FIT interactors show a link with root cell elongation (e.g. EIN3/EIL1, Mediator, polycomb, and DELLA). In the future, it will be interesting to investigate roles and molecular mechanisms cross-connecting –Fe, ABA, and root cell elongation responses via FIT.

Taken together, by exploiting information from –Fe co-expression clusters and FIT-dependent and -independent gene signatures, we integrate new components in regulatory cascades. Fe responses may have a far more drastic importance in stress and root developmental responses than previously thought. Cross-connections of FIT with environmental and developmental signaling pathways in regulatory hubs suggest that root Fe acquisition can be uncoupled from Fe homeostasis regulation at the whole-plant level.

## Supplementary data

Supplementary data are available at *JXB* online.

Table S1. List of genes in the root –Fe co-expression network (Fig. 1) and FIT-dependent and FIT-independent expression patterns in *Arabidopsis thaliana.*

eraa012_suppl_supplementary_table_S1
